# Fat Graft Size: Relationship Between Cannula and Needle Diameters

**DOI:** 10.7759/cureus.7598

**Published:** 2020-04-09

**Authors:** Oscar A Vazquez, Moses I Markowitz, Hilton Becker

**Affiliations:** 1 Surgery, Charles E. Schmidt College of Medicine, Florida Atlantic University, Boca Raton, USA; 2 Molecular, Cellular, and Developmental Biology, University of Michigan, Ann Arbor, USA

**Keywords:** fat, fat grafting, cannula, plastic surgery

## Abstract

Background

It is generally believed that trauma to fat grafts is detrimental and affects the survival of the graft. In addition, it has been shown that smaller fat particle size corresponds to better survival; however, smaller cannula openings correspond to slower and more difficult fat graft harvesting.

Objectives

This study documents the relationship between cannula size, harvested fat cell size, and injection needle size. A means of reducing fat particle size following aspiration with larger diameter cannulas is also discussed.

Methods

Fat was harvested from five patients undergoing elective liposuction. Each fat sample was placed in a syringe and injected through progressively smaller needles until obstruction under low pressure was obtained. The minimal needle size was documented for each sample.

Results

Fat harvested with a liposuction cannula results in different size fat particles ranging up to the size of the cannula. Particles obtained from 3- and 4-mm cannulas can be injected without obstruction through a 16-gauge needle. Particles obtained from a 2-mm cannula can be injected without obstruction through an 18-gauge needle. Particles obtained from a 1-mm cannula can be injected without obstruction through a 20-gauge needle. Particles obtained from a 1-mm cannula could not be injected without obstruction through a 22-gauge needle.

Conclusions

There is a relationship between cannula opening size and the resultant fat graft size. Fat particles are somewhat compressible but should not be forced through needles or cannulas that are too narrow. It may be beneficial to harvest fat with larger cannulas and cut the particles to smaller sizes for injection.

## Introduction

Fat grafting is a commonly performed procedure, and while many different techniques are used, data typically show inconsistent fat graft survival rates [1]. There are many factors that claim to enhance fat graft survival, including the donor site, preparation of the recipient site, aspiration pressure, use of lidocaine, addition of platelet-rich plasma, fat graft preparation (e.g., centrifugation, straining, decanting, and various devices to prepare the fat), cannula size, needle size, injection technique used, and volume of injection. Of these factors, there has been a focus on harvesting and injecting fat without trauma as these are shown to be important for fat cell survival [2-4]. Fat graft size, stromal vascular fraction (SVF), and stem cell enhancement have also all been shown to be beneficial to fat graft survival in the long term [1,5]. Kirkham et al. found that larger harvesting cannulas facilitate the collection of larger fat particles and better adipocyte viability, with greater overall volume retention [5]. Studies also show that fat grafts larger than 2 mm in diameter according to Khouri et al. and 1 mm in diameter according to Kato et al. will survive less [6,7]. Particles of fat greater than 1 mm in diameter will suffer necrosis due to insufficient blood supply to the inner core. This is due to smaller particles having an increased surface area that is exposed to the tissue fluid, resulting in a greater survival rate [8]. In this study, we investigated the fat particle size harvested with a set diameter cannula that was passed without trauma through a specific needle internal diameter.

## Materials and methods

Fat was harvested from five patients undergoing elective liposuction. The fat was harvested under the same pressure using a standard set of cannulas. Investigational cannulas were specifically manufactured with known internal diameters ranging from 1 to 4 mm. Each cannula had a single opening, with the same size as the internal diameter. The fat particles obtained from these samples were examined under Canon photography magnification in the operating room after extraction and were photographed. Each fat sample was then placed in a syringe and injected through progressively smaller needles until obstruction under low pressure was obtained. The minimal needle size was documented for each sample. In this measurement, 16-, 18-, 20-, and 22-gauge needles were used.

## Results

**Figure 1:**
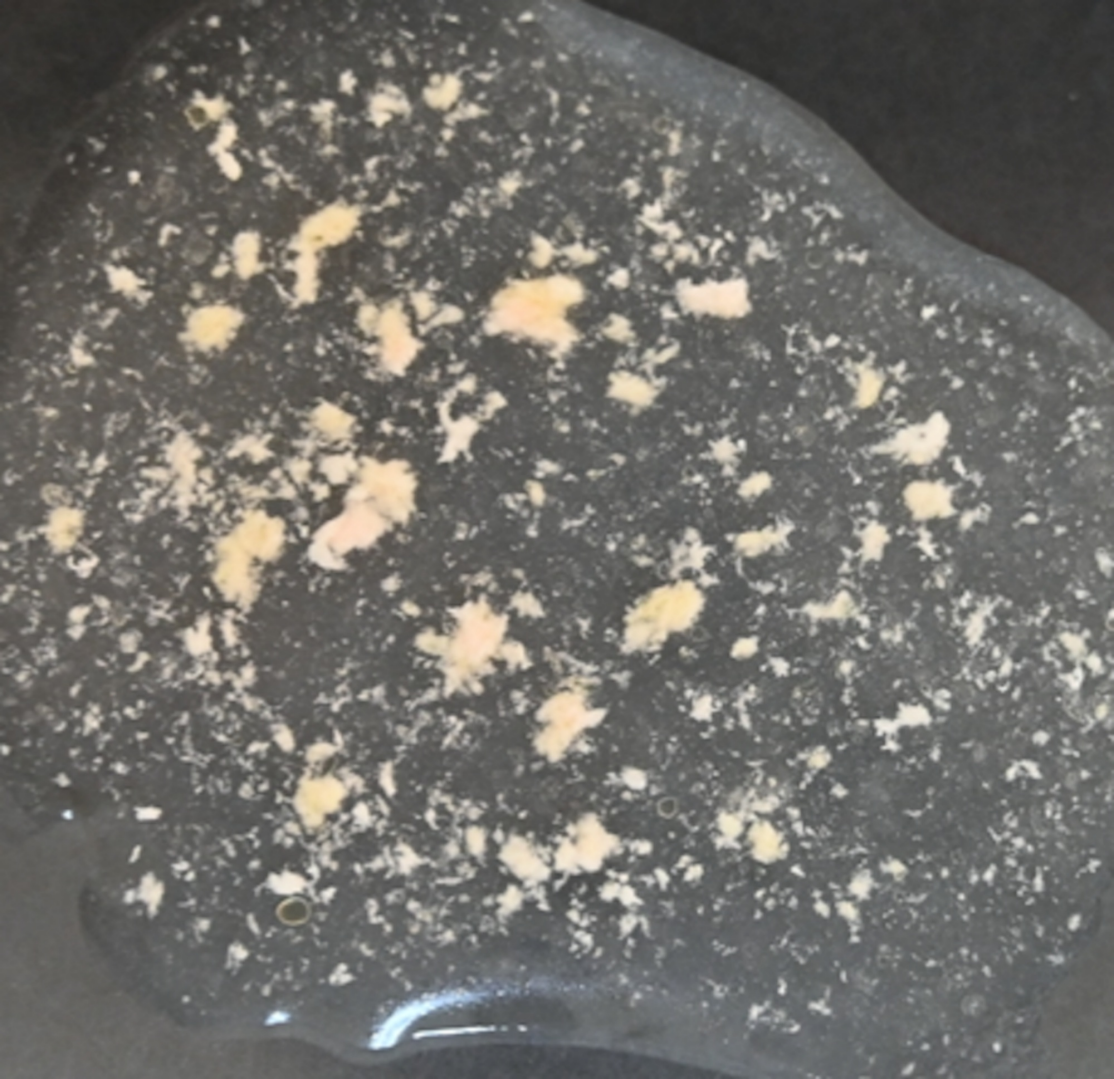
Fat harvested with a 4-mm cannula produced a variety of particle sizes.

**Figure 2:**
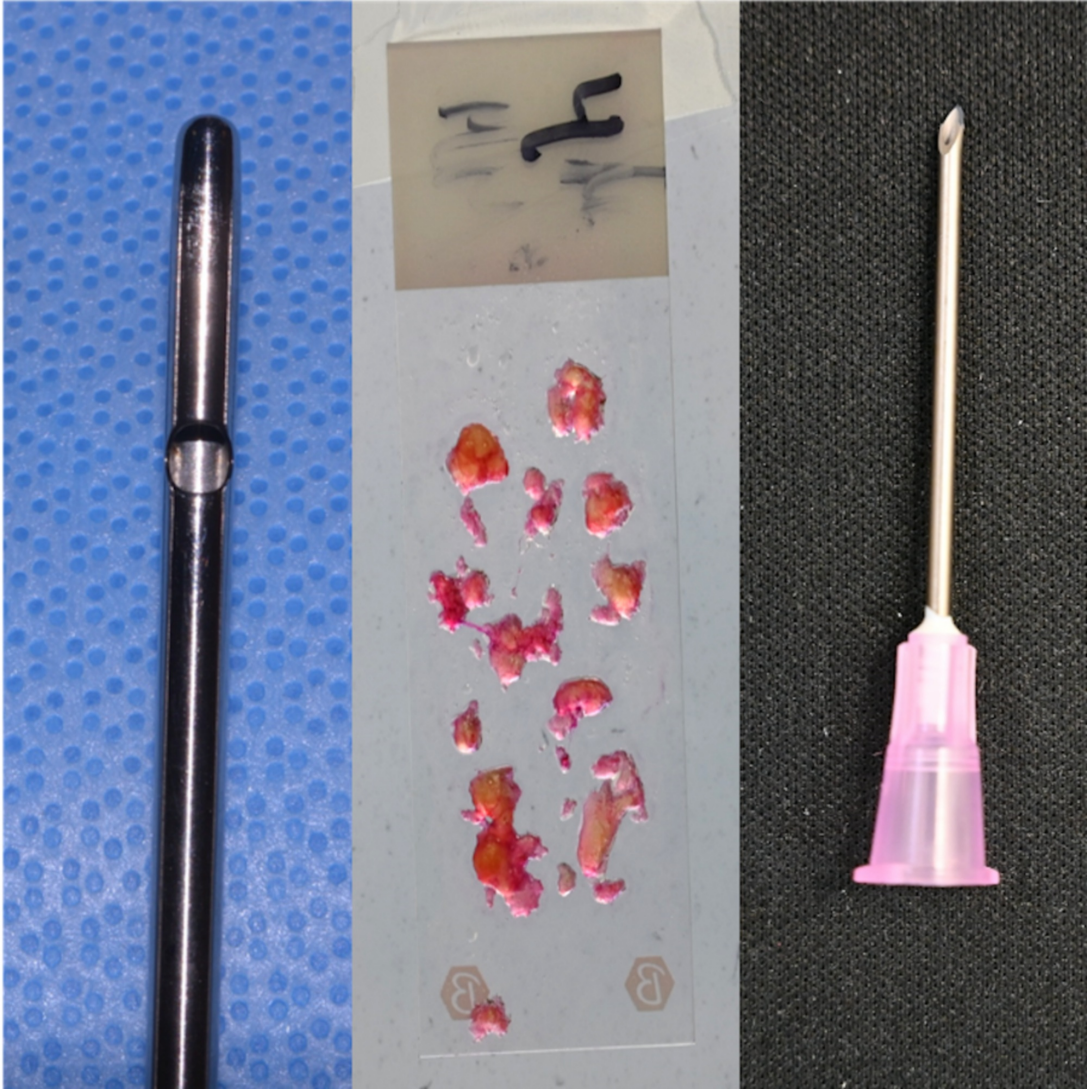
Fat harvested with a 4-mm cannula produced particles up to 4 mm in size, which were successfully injected without obstruction through a 16-gauge needle with a nominal internal diameter of 1.19 mm.

**Figure 3:**
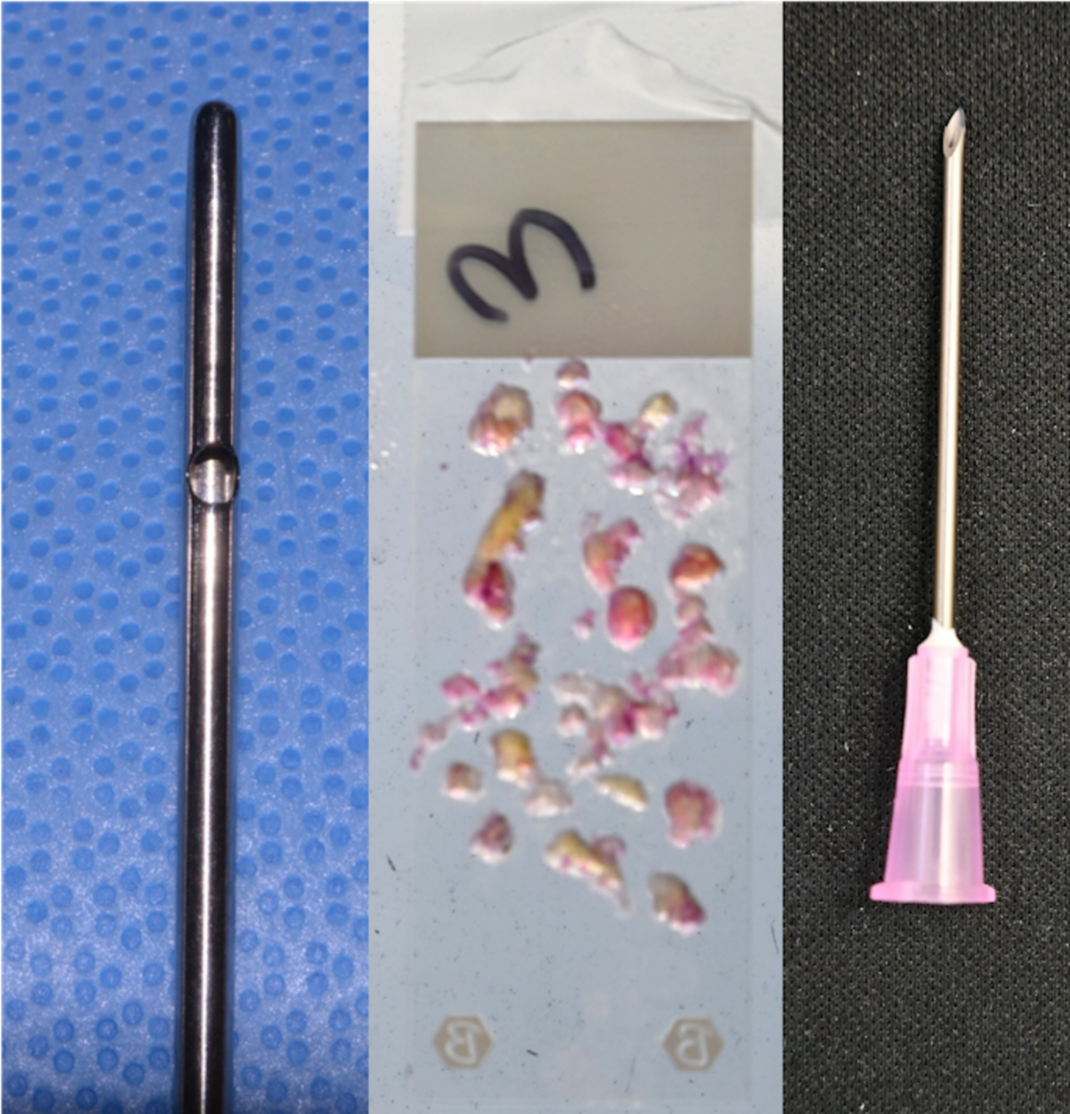
Fat harvested with a 3-mm cannula produced particles up to 3 mm in size, which were successfully injected without obstruction through a 16-gauge needle with a nominal internal diameter of 1.19 mm.

**Figure 4:**
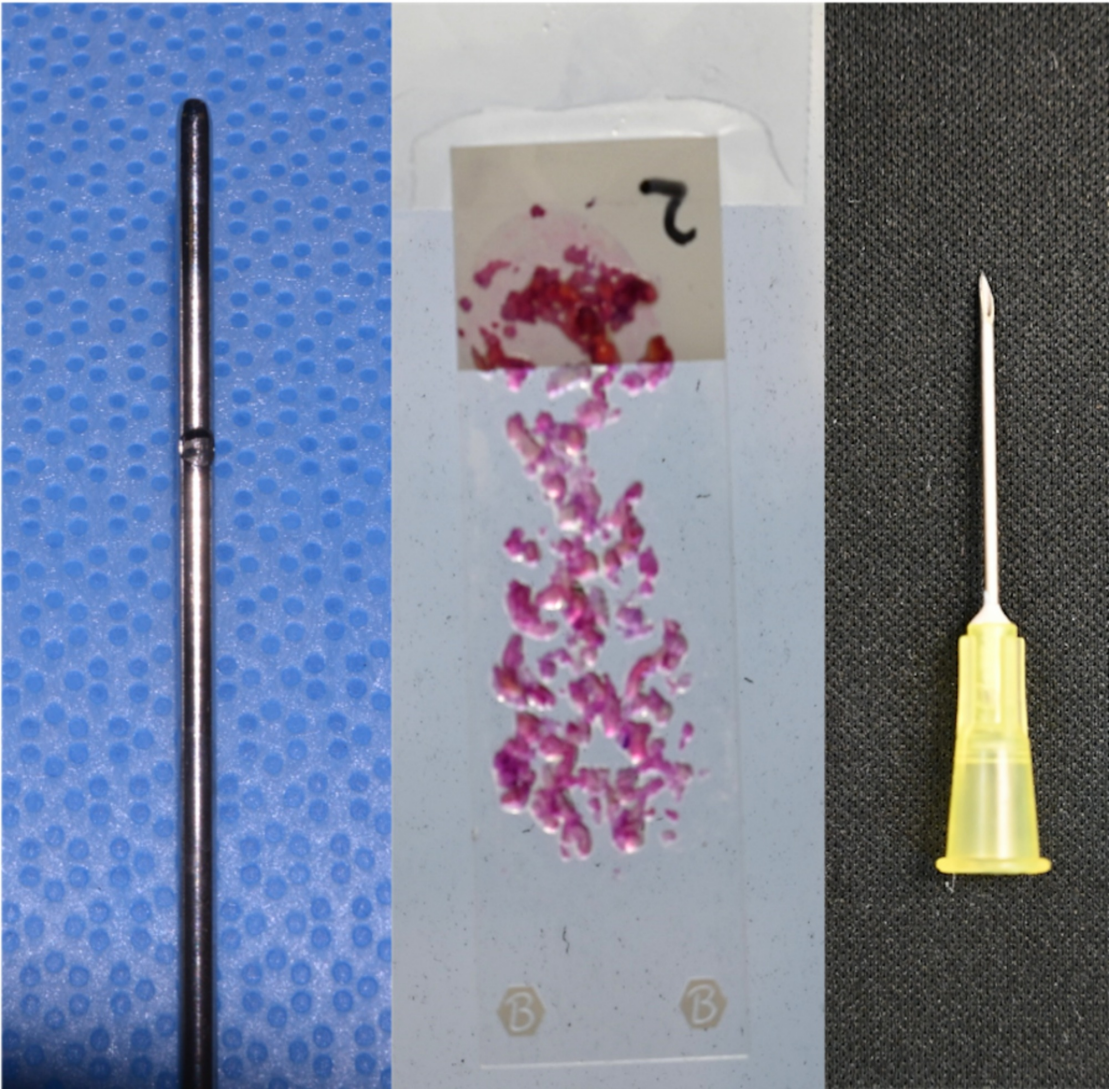
Fat harvested with a 2-mm cannula produced particles up to 2 mm in size, which were successfully injected without obstruction through an 18-gauge needle with a nominal internal diameter of 0.84 mm.

**Figure 5:**
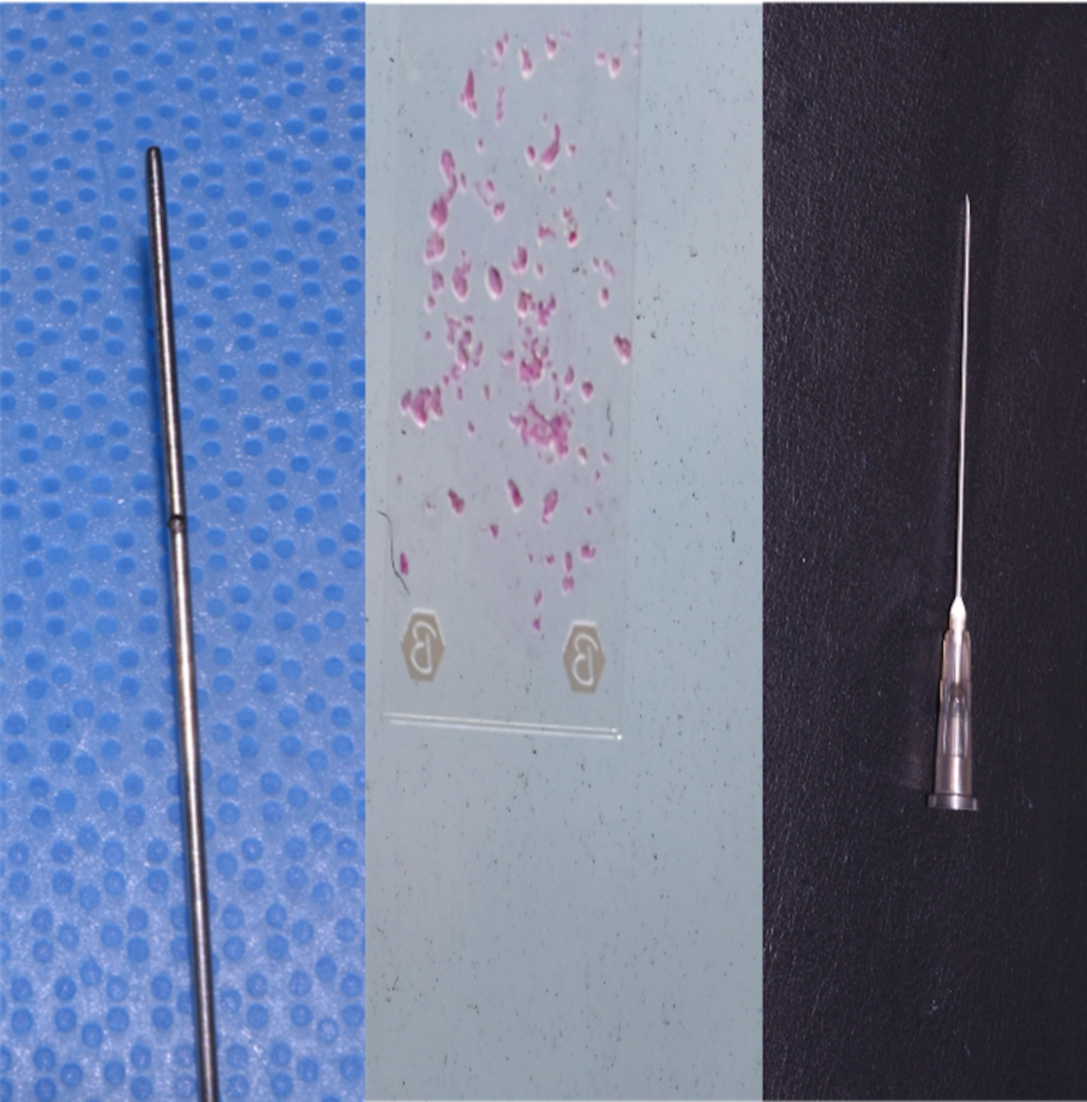
Fat harvested with a 1-mm cannula produced particles up to 1 mm in size, which were successfully injected without obstruction through a 20-gauge needle with a nominal internal diameter of 0.60 mm.

Fat harvested with a 4-mm cannula resulted in numerous particles of various sizes (Figure 1). As fat particles are compressible to a certain degree; 4- and 3-mm particles can be injected without obstruction through a 16-gauge needle (nominal internal diameter: 1.19 mm) (Figures 2, 3). Particles of 2 mm in size can be injected without obstruction through an 18-gauge needle (nominal internal diameter: 0.84 mm) (Figure 4). Particles of 1 mm in size can be injected without obstruction through a 20-gauge needle (nominal internal diameter: 0.60 mm) (Figure 5). Particles of 1 mm in size could not be injected without obstruction through a 22-gauge needle (nominal internal diameter: 0.41 mm).

## Discussion

In 1919, Marchand conducted a study to investigate the viability of larger fat transplantations. The study demonstrated that regions of the graft greater than 2 mm from the edge of the graft underwent necrosis due to insufficient vascularization of the tissue by around the 10th week post-grafting [9]. This is further supported by mathematical modeling by Khouri et al., placing this limit at around 1.6 mm from the closest blood supply [6]. Histological analysis of fat grafting demonstrated that cells will not survive if they are deposited greater than 1.5 ± 0.5 mm from the host tissue. For this reason, a size of 2 mm is recommended as a conservative upper limit under ideal circumstances [10]. Additionally, Kato et al. published a study demonstrating that fat particles are made up of three layers: a layer of mitotic cells bordered by a surviving outer layer, with a necrotizing inner core [7]. To ensure this dispersion is performed effectively, current practices use smaller syringes and smaller gauge needles for injection.

Rohrich et al. found no significant difference in cell viability in adipose tissue removed from the abdomen, flank, thigh, and knee with handheld syringe aspiration, and Small et al. found no difference in volume retention in fat grafts formed from different donor sites [11,12]. In terms of the recipient site, mobile areas of the face, such as the glabella and lips, are less amenable to correction compared with less mobile areas such as the malar and lateral cheek [13]. Studies investigating the effect of the recipient site on fat grafting and retention have been inconclusive [14]. When fat is grafted to the torso or breasts, a 3- to 5-mm cannula is commonly used; larger particles of fat (2 to 3 mm) are then aspirated. The limiting factor for larger grafts is the syringe opening, which is 2 mm in size. When fat is grafted in the face, smaller particles are required that are often less than 1 mm in size. It has therefore been suggested that fat be harvested with a cannula having a 1-mm opening [15].

**Figure 6:**
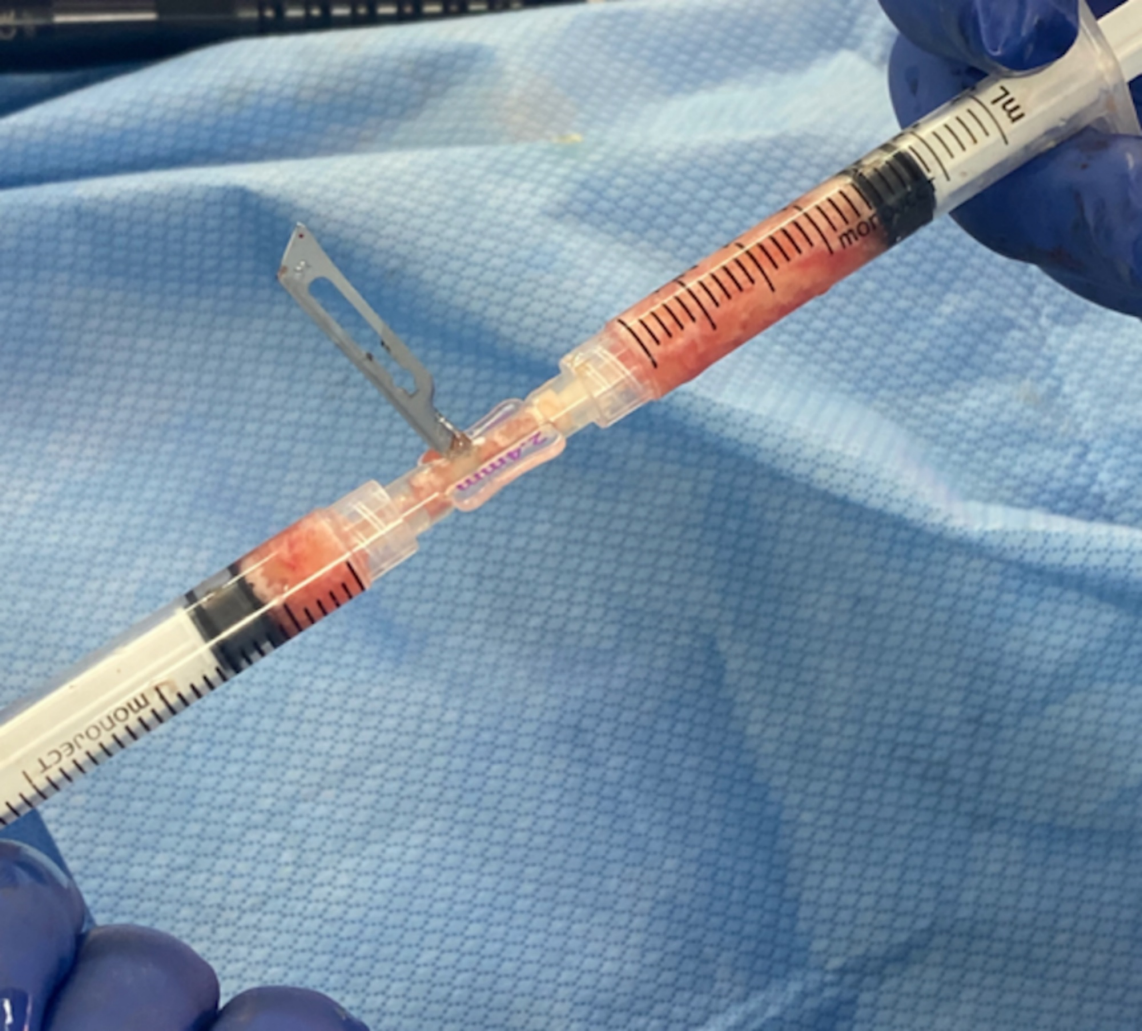
Blade in a Luer-to-Luer connector.

The standard Coleman technique involves harvesting fat with a 3-mm cannula and injecting with a 1.5-mm cannula [16]. Currently, micro-fat injections are performed with a 1- or 0.7-mm cannula. The SNIF (sharp-needle intradermal fat grafting) procedure is advocated to be performed with a 23-gauge needle with a nominal inner diameter of 0.33 mm [15]. This needle often becomes blocked as the fat particles harvested with a 1-mm cannula result in fat particles larger than the inner diameter of the needle. If a larger diameter cannula (e.g., 3 to 5 mm) is used for fat harvesting, a Luer blade can be used to cut the larger fat particles into smaller particles (Figure 6). These smaller fat particles can then be injected without trauma through narrower gauge needles. It is postulated that fat particles harvested from larger cannulas will contain more SVF, which will then be retained in the smaller fat particles when reduced in size [3]. This study highlights the relationship between the fat particle size obtained from a harvesting cannula internal diameter and the injection needle size. For fat grafting procedures, we recommend surgeons consider the following:

1. The internal diameter of the cannula and the diameter of the cannula openings (if more than one) and how these relate to the resultant fat particle sizes.

2.The internal diameter of the injection cannula or needles relative to the fat particle sizes.

3. Fat harvested with a liposuction cannula results in different size fat particles ranging up to the size of the cannula.

4. Standard injection syringes have an opening of 2 mm; therefore, fat harvested with a cannula greater than 3 mm should not be injected through a standard syringe.

5. Although fat particles are somewhat compressible, fat should not be injected forcibly through needles or cannulas as this results in trauma to the fat cells. Furthermore, injecting under pressure results in an increased risk of intravascular injury.

6. Larger fat particles can be cut into smaller particles for injection with finer needles using a Luer blade.

There has been a substantial increase in research interest to identify methodologies for optimizing fat graft survival. Despite some differences in harvest and implantation techniques in the laboratory, these findings have not translated into a universal protocol for fat grafting [14]. If fat is harvested with a larger cannula, the fat particles will undergo damage when injected through a narrower needle or cannula, thus reducing their viability. As surgeons move toward injecting fat through smaller cannulas and needles, it is important to be aware of the size of particles being injected through these needles. If fat particles are forced through narrower needles, they are subject to undue trauma, reducing their viability. This makes injection far less precise and damages the transplanted tissue.

## Conclusions

There is a relationship between the cannula internal diameter and the resultant fat graft size. Therefore, it is important to know the size of fat particles harvested and the appropriate needle size for atraumatic injection. In order to reduce the trauma to the fat particles, we theorize that fat should not be forced through needles or cannulas that are too narrow.
